# Influence of Landing in Neuromuscular Control and Ground Reaction Force with Ankle Instability: A Narrative Review

**DOI:** 10.3390/bioengineering9020068

**Published:** 2022-02-10

**Authors:** Jian-Zhi Lin, Yu-An Lin, Wei-Hsun Tai, Chung-Yu Chen

**Affiliations:** 1 Department of Physical Education, National Taiwan University of Sport, Taichung 40404, Taiwan; jzlin@ntus.edu.tw; 2 Department of Physical Education, National Taiwan University, Taipei 10610, Taiwan; 3 School of Physical Education, Quanzhou Normal University, Quanzhou 362000, China; taidavid@qztc.edu.cn

**Keywords:** ankle sprain, unanticipated landing, dynamic tasks, electromyography

## Abstract

Ankle sprains are generally the most common injuries that are frequently experienced by competitive athletes. Ankle sprains, which are the main cause of ankle instability, can impair long-term sports performance and cause chronic ankle instability (CAI). Thus, a comprehensive understanding of the key factors involved in repeated ankle strains is necessary. During jumping and landing, adaptation to the landing force and control of neuromuscular activation is crucial in maintaining ankle stability. Ankle mobility provides a buffer during landing, and peroneus longus activation inhibits ankle inversion; together, they can effectively minimize the risk of ankle inversion injuries. Accordingly, this study recommends that ankle mobility should be enhanced through active and passive stretching and muscle recruitment training of the peroneus longus muscles for landing strategies should be performed to improve proprioception, which would in turn prevent ankle sprain and injury to neighboring joints.

## 1. Introduction

**Figure 1:**
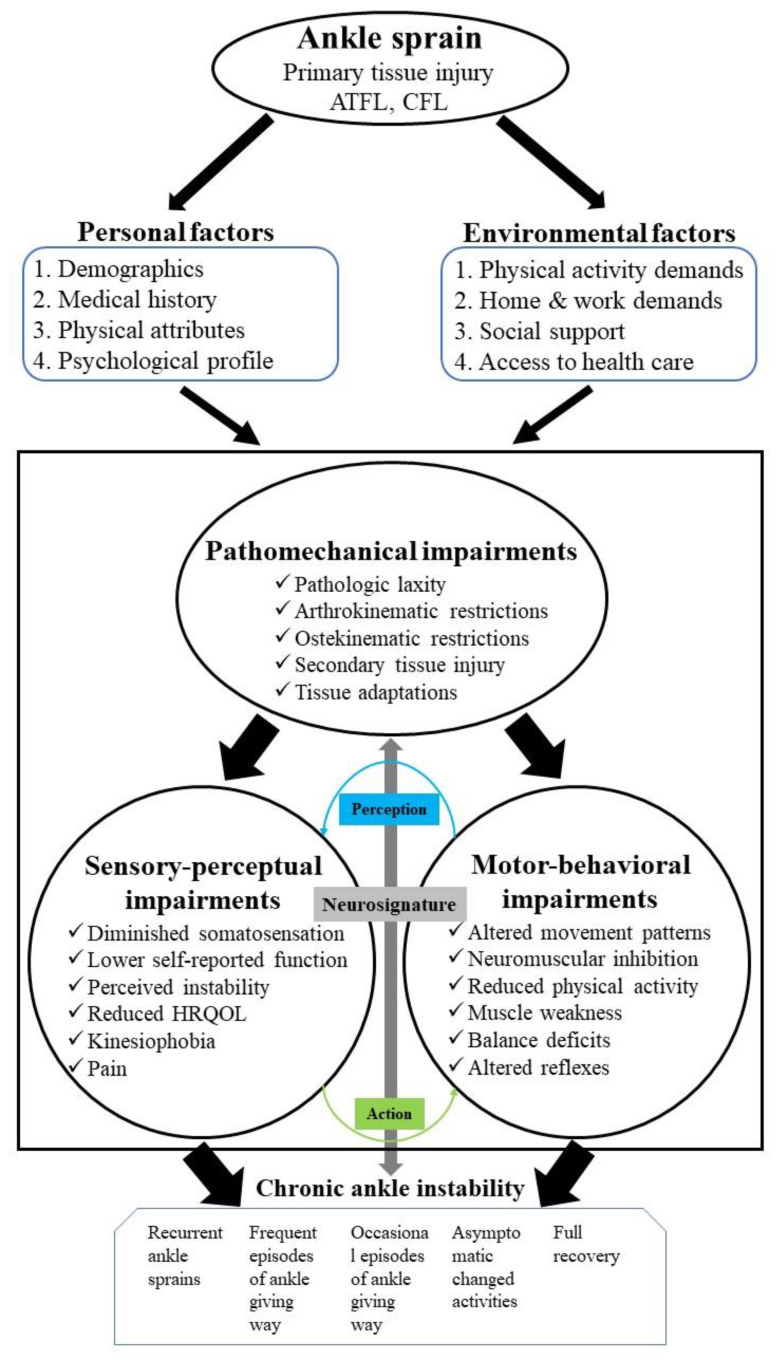
The updated model of chronic ankle instability. Note: CAI is determined at least 12 months after the initial ankle sprain; ATFL: Anterior talofibular ligament; CFL: Calcaneofibular ligament; HRQOL: Health-related quality of life; Modified from Hertel and Corbett (2019).

Competitive athletes often experience lower extremity musculoskeletal injuries during sport and military style physical activities, particularly lateral ankle sprains (LAS) [1,2,3,4]. Studies indicate that 40–75% of people who experienced LAS for the first time developed chronic ankle instability (CAI) due to pathomechanical impairments, sensory–perceptual impairments, or motor behavioral impairments. The updated model of CAI is usually characterized by eight primary factors, including: (1) primary tissue injury; (2) pathomechanical impairments; (3) sensory–perceptual impairments; (4) motor behavioral impairments; (5) personal factors; (6) environmental factors; (7) component interactions; (8) the spectrum of clinical outcomes (Figure 1) [1,5,6,7,8]. Its main symptoms include repetitive episodes or perceptions of the ankle giving way, ongoing symptoms such as ankle pain, partial functional impairment, weakness, structural impairment, proprioception, and reduced ankle range of motion (ROM) [1,9,10]. CAI is associated with decreased neuromuscular control, thus aggravating ankle instability and further reducing proprioception. Therefore, adjustments must be made in the lower extremity kinetic chain while landing to decrease force transfer and impact on the lower extremities [11,12,13,14].

When performing dynamic tasks such as cutting, jumping, and landing movements, the ankles exhibit inversion and/or plantar flexion, internal rotation, and foot adduction (calcaneal supination), which can result in ankle sprain injuries [15,16,17], anterior cruciate ligament (ACL) injuries, and other lower extremity-related injuries [18]. Two studies indicated that ankle sprains may be related to knee injuries, based on a significant association between ankle sprain history and knee injury history [19,20,21]. As reported, an excessive ground reaction force (GRF) during landing can, in a non-contact situation, increase knee abduction and ankle supination, thereby increasing the risk of lower extremity injuries [22,23]. Accordingly, using unfavorable coordination strategies to perform movements may greatly increase the risk of ankle injuries [24]. In most jumping tasks, the activation levels of the muscle groups surrounding the lower extremity joints and the amount of GRF are adjusted from the distal to the proximal end. Inappropriate adjustment of the two mechanisms can largely reduce their ability to protect the knee joints, resulting in ACL injury and worsening ankle instability [25].

Theisen and Day [14] observed that people with CAI had different movement patterns in the lower extremities, particularly, a smaller knee flexion angle, than those without CAI. Having an ideal joint mobility is conducive to absorbing the force buffer caused by the external environment. However, when joint mobility is limited, favorable landing patterns can only be achieved through the musculoskeletal system [26]. Joint stability depends on the interaction between active (muscles and proprioception) and passive (joint capsule and ligament) tissues [27]. The muscles are activated to provide a dynamic defense mechanism [28]; for example, the peroneus longus and tibialis anterior muscles stabilize the ankles [29]. Because an ankle sprain greatly reduces ankle stability, the ankles must be stabilized when performing dynamic tasks through coordination and control mechanisms among other lower extremity joints [30]. The present literature review assesses the characteristics of GRF and neuromuscular activation in people with ankle instability; this allows understanding the movement control strategies and muscular activation control following an ankle sprain and provides coaches, athletes, and clinicians with insights into ankle instability and risk assessment of other lower extremity injuries. In this work, comprehensive literature and original and review articles searches were performed to identify peer-reviewed journal articles on lower extremity muscle activation or GRF during landing with ankle instability. Two independent authors (Lin, J.Z. and Lin, Y.A.) systematically searched the literature in electronic databases. The online databases of PubMed, Web of Science, SPORTDiscus, and CINAHL were searched from inception through to 2021 using medical subject headings, vocabulary, and keyword searches. The keywords included terms such as (1) ankle instability or CAI or ankle sprain; (2) biomechanics or electromyography or EMG; (3) peroneal longus or tibialis anterior or gastrocnemius; (4) landing; (5) English language. Duplicate studies were excluded. Inclusion criteria: all articles were considered for inclusion, irrespective of their publication date. Studies were excluded if (1) the full text was unavailable; (2) the text was not written in English; (3) the text did not mention ankle instability, sprain, and CAI or muscle; (4) the text was deemed irrelevant by the authors. Exclusion criteria: articles were excluded if (1) they investigated initial ankle sprain injury; (2) used ankle bracing on landing; (3) examined kinematic, kinetic, and/or muscle activity variables during bilateral jumping; (4) fatigue on landing; (5) the articles were not published in a peer-reviewed journal.

## 2. The Importance of Neuromuscular Control for Ankle Sprains

**Figure 2:**
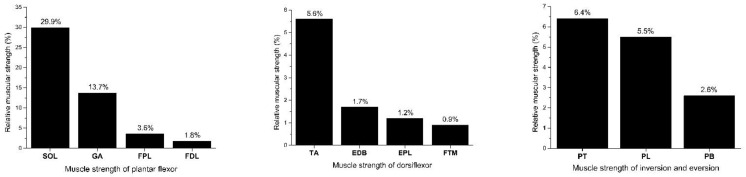
Relative muscle strength percentage of ankle (%). Note: SOL: Soleus; GA: Gastrocnemius; FPL: Flexor pollicis longus; FDL: Flexor digitorum longus; TA: Tibialis anterior; EDB: Extensor digitoralis longus; EPL: Extensor pollicis longus; FTM: Fibularis tertius muscle; PT: Posterior tibial; PL: Peroneal longus; PB: Peroneal brevis; Modified from Nordin and Frankle (2001).

The ankle comprises approximately 28 bones, including the sesamoid bones in the foot; the main bones are the tibia, fibula, talus, and calcaneus. These bones, in cooperation with the neighboring ligaments and muscle groups, provide satisfactory joint mobility and a complete range of joint movements in three axes: dorsiflexion and plantar flexion, inversion and eversion, and pronation and supination [31]. The mechanisms of ankle injuries include bone structure, ligament strength, muscular activation, and movement coordination. From the perspective of the benefits of lower extremity muscle activation, this study first explored the prime movers of the ankle: the plantar flexors, invertors, and evertors. According to the relative strength of ankle muscles (Figure 2) [31], the largest contributor among the plantar flexors is the soleus, the tibialis anterior muscle among the dorsiflexors, the tibialis posterior muscle among the invertors, and the peroneus longus muscle among the evertors, which contribute the most to ankle sprain resistance [32,33,34].

Electromyography (EMG)—a common research instrument used to conduct neuromuscular activation analysis in relation to ankle instability—involves observing muscular activation characteristics before and after landing from a jump. EMG data are represented using the average, root mean square, and integral value [35,36,37], all of which can be standardized on the basis of maximum voluntary isometric contraction (or maximum voluntary contraction from the maximum value of the task) to facilitate a comparison between different movements or muscles [38,39]. In summary, EMG serves as a crucial indicator for assessing neuromuscular control capacity, reveals the level of activation and contribution of muscle groups surrounding the ankles during landing, and offers a complete description of the relationships between joints and muscles.

The mechanism of an ankle sprain involves over-inversion and/or foot adduction (calcaneal supination), may or may not present plantar flexion [40,41], and includes the impact of external forces during landing; an ankle sprain occurs easily when the peroneus longus muscle cannot prevent inversion in a timely manner [25,42]. Suda et al. [43] conducted a study of 21 people with functional ankle instability (FAI) and 19 control group participants who were asked to perform vertical jump smashes with both feet. They analyzed the activation of the tibialis anterior, gastrocnemius, and peroneus longus muscles 200 ms before and after landing and found that those with FAI exhibited a significantly lower level of peroneus longus muscle activation 200 ms before landing and a significantly higher level of tibialis anterior muscle activation 200 ms after landing than the control group [43]. The researchers also investigated neuromuscular control in people with CAI performing jumping and landing movements, which yielded consistent results [44]. These findings verified that people with ankle instability sustained partial damage to their neuromuscular control due to a history of inversion sprains, and this damage could reduce the level of peroneus longus activation or increase the reaction time [42,45,46]. These subjects’ movement patterns changed after the sprains, persisting even after ≥3 months of complete recovery from the sprain. The most direct consequence of sprains is the incomplete activation of the peroneus longus muscle before landing, which has long been considered key to preventing recurrent ankle sprains. Studies have shown that the subtalar joint supinates before landing [36,47], and the peroneus longus activation, which stabilizes the ankle, positions the subtalar joint from the inverted position back to the neutral position when the foot lands on the ground [43,48,49]. In the presence of proprioception or neuromuscular impairment, people with ankle instability will exhibit limited peroneus longus pre-activation; in addition, ankle sprains typically occur in unexpected situations. Therefore, to prevent ankle sprains, the subtalar joint must be in the neutral position, and the peroneus longus muscle must be activated in a timely manner.

According to Kipp [30], to combat ankle instability following an ankle sprain, a coordination and control strategy known as the compensatory mechanism, among other joints, is induced to keep the joint stable [30]. The compensatory mechanism in people with ankle instability involves a high level of activation of the proximal joint muscles, such as muscle groups surrounding the ankle, knee, and hip joints, to compensate for incoordination caused by ankle instability, thus stabilizing the kinetic chain in the lower extremity. For example, the gastrocnemius and rectus femoris muscles play a critical role in landing from a jump [50,51]. The two muscles are the concurrent prime movers of two joints each; the gastrocnemius muscle serves as the ankle extensor and knee flexor, and the rectus femoris muscle serves as the knee extensor and hip flexor. The two muscles act to reduce ankle or knee injuries [52]. Overall, neuromuscular control exerts a great influence on ankle sprains. For people with CAI, the quality of their joint movement patterns and muscular activation is compromised to some extent; however, they can still perform high-intensity exercises with satisfactory coordination and control strategies among their joints and muscles. Accordingly, in addition to facilitating peroneus longus strengthening to train muscle recruitment and reduce the occurrence of inversion sprains, the muscle groups surrounding other joints must be enhanced to provide favorable landing patterns for people with CAI.

## 3. The Influence of Landing Force on Ankle Instability

Most studies have investigated the effect of ankle instability on GRF during landing; these studies analyzed the peak vertical GRF [36,53,54,55,56,57], peak anterior and posterior GRF [36,53], peak medial and lateral GRF [36,53,57], and the time to peak [36,47,53,54,57,58]. A consistent finding was that people with ankle instability had a significantly higher peak vertical GRF and a greater difference between the times of landing. Accordingly, the landing force is a high-risk injury indicator for people with ankle instability [15,54,59,60]. The landing force is closely associated with motor coordination. An unfavorable motor coordination results in an excessively high landing force and a low buffering effect, leading to excessive load on the feet during landing and damaging the lower extremity muscular system [61,62]. This is one of the main causes of ankle instability [63,64]. The lower extremity joints form a closed kinetic chain during landing, and the collision load from the vertical GRF is transferred through the ankle, knee, and hip joints to the proximal joints [26,61]. In this continuous process, and in the context of unfavorable neuromuscular control coupled with ankle instability, the risk of sport injuries in athletes is markedly increased. The amount of landing force is affected by external conditions [62]. A study revealed that jumping down vertically from a 30 cm-high platform will yield a landing force four times higher than the jumper’s body weight [65]. Attenborough et al. and Bates et al. argued that an excessively high vertical GRF during landing may result in ankle or knee instability. Therefore, vertical GRF has been a common indicator adopted by researchers to assess load on lower extremity joints.

The buffering movement performed by humans in response to GRF is mostly limited to the movement range of the ankle. De Ridder et al. [54] investigated the multi-segment foot landing kinematics in 38 subjects with CAI, 28 copers, and 30 controls. The results showed that the CAI and coper groups exhibited less ankle plantar flexion at touchdown. Additionally, the study demonstrated that the total ROM of the ankle in the sagittal plane decreased more in people with CAI than in the control group [54]; such a smaller movement range can result in a stiffer landing, causing the vertical GRF and landing load to soar [54,66]. A limited ankle dorsiflexion affects how people absorb the landing force with the gastrocnemius–soleus complex, resulting in increased stress to the talar articular surface [67]. According to Brown et al. [53] people with LAS sustain a high level of vertical GRF and loading rate. This is possibly because repeated sprains lead to the deterioration of the articular cartilage and to osteoarthritis [68], which undermines the resistance of the articular cartilage to impact, rendering it unable to react to the rapid and high load collision force during landing and thus creating high-risk factors in the transfer of force to the ankle [53,68]. If an invasive medical treatment is not performed on people with ankle instability, an examination must be performed to determine whether they have developed osteoarthritis in the ankle, because chronic ankle conditions are directly related to the effects of the vertical landing force and the loading rate during running [25,60]. CAI subjects also showed decreased ROM of the ankle and decreased time to peak GRF during a single leg jump landing compared to healthy controls. This evidence indicates that CAI makes it improbable to attenuate the landing force and increases the stress transmitted to the ankle joint [47]. Accordingly, people with ankle instability have an unfavorable buffering effect on the vertical load and the time of muscle action due to injuries in the proximal joints, which in turn changes the movement patterns and even creates a unique compensatory strategy. In the long run, this may increase the risk of injury to other joints in the lower extremity kinetic chain [16,69].

## 4. How CAI Patients Show Postural Control Deficits during Landing Tasks

From a movement control perspective, the primary factors that affect motor reactions in an unanticipated landing task are the allocation of attention and the time available to react. The main stimulation stage for the allocation of attention occurs before the performance of a movement until the completion of the movement task. The available time to react refers to the performance result of an immediate reaction movement pattern affected by the attention allocation [58,70]. However, landing tasks are usually unanticipated in most real-world sports scenarios. Athletes who developed LAS or ACL injuries typically focused their attention on the hand, ball, or a particular target during landing [18,70,71]. This indicates that when people with ankle instability perform an unanticipated movement, more sensory information is required in lower extremity activities to stimulate neuromuscular reactions. Static and dynamic postural control deficits are consistently found in individuals with acute LAS and CAI [72,73,74,75,76,77,78]. Based on the current data, evidence indicates that CAI exists in subjects with postural control impairments. After an ankle injury, proprioceptive and neuromuscular insufficiencies alter postural control, leading to different compensatory strategies. Therefore, constraints of the sensory system, motor system, and central nervous system may lead to alterations in lower extremity joints and subsequently altered joint loading [67]. Accordingly, sensory information stimulation is greater in gait than in an unanticipated landing. When people with ankle instability perform an unanticipated landing, functional ankle instability (FAI) and the lack of stimulation by sensory information may result in inadequate lower extremity muscle activation, thus increasing the risk of injury during landing.

The ideal balancing movement during landing involves integration among the sensory system, central nervous system, and motor system [79] (Table 1). The inner ear’s vestibule, the optic verve, and proprioception in the sensory system maintain the center of mass of the body in the correct base of the support area. The central nervous system integrates and coordinates limb movements, muscular activation, and balancing strategy. Finally, the motor system generates the main movement pattern and dynamically modifies the limb positions to maintain the center of gravity in a balanced and stable state [80,81,82]. Stabilizing and balancing in a dynamic posture requires the integration of involuntary sensations and impulses and precise muscle recruitment to control the level of muscular activation to perform appropriate and coordinated movements [83].

In terms of information processing, balancing strategies in human body movements usually involve the use of automatic processing to respond to external interferences; automatic processing refers to the unconscious automatic execution of strategies through rapid and direct information processing, which requires no attention and is not disturbed by the external environment [84]. Common stabilizing strategies include the ankle strategy, knee strategy, hip strategy, stepping strategy, and suspensory strategy. The lower extremity musculoskeletal system generates continuous coping strategies to stabilize the limbs when its movements are impaired. Increasing joint stability is among the most critical balancing strategies. During jumping and landing, the lower extremity is loaded with body weight and needs to act against the load from the height of the jump. Because static restraints such as the articular capsule and ligament cannot withstand such external load, the dynamic restraints come into play to maintain the dynamic stability of the limbs; this working process of the dynamic restraints is known as dynamic joint stability [85].

## 5. Conclusions

Studies investigating neuromuscular control and GRF in people with ankle instability have revealed that the main muscular control problem of patients or athletes with recurrent ankle sprains is insufficient peroneus longus activation. For people with an inadequate buffer against the landing force, the presence of chronic joint conditions must be determined through relevant examinations, including an ankle mobility assessment. The timely performance of active or passive stretching also helps increase the range of motion. With regard to overall injury prevention strategies, muscle recruitment training of the peroneus longus muscle should be performed to develop an appropriate landing strategy; strengthening the peroneus longus activation can further prevent recurrent ankle sprains and the occurrence of other lower extremity injuries.

## Figures and Tables

**Table 1:** Balance control system of posture stability.

System	Content
Sensory system	Vision, vestibular sense, proprioception, touch, vibration sense
Motor system	Muscle strength, neuromuscular control
Central nervous system	Integration of sensory and motor factor

## Data Availability

Not applicable.
